# Weld Seam ROI Detection and Segmentation Method Based on Active–Passive Vision Fusion

**DOI:** 10.3390/s25247530

**Published:** 2025-12-11

**Authors:** Ming Hu, Xiangtao Hu, Jiuzhou Zhao, Honghui Zhan

**Affiliations:** 1 School of Electrical Engineering and Automation, Anhui University, Hefei 230601, China; ahu_hoo1999@163.com; 2 School of Future Technology, Huazhong University of Science and Technology, Wuhan 430074, China; 3 Wuxi Research Institute, Huazhong University of Science and Technology, Wuxi 214000, China

**Keywords:** weld grinding, weld inspection, point cloud segmentation, active–passive vision fusion, YOLOv8n-seg

## Abstract

Rapid detection and precise segmentation of the weld seam region of interest (ROI) remain a core challenge in robotic intelligent grinding. To address this issue, this paper proposes a method for weld seam ROI detection and segmentation based on the fusion of active and passive vision. The proposed approach primarily consists of two stages: weld seam image instance segmentation and weld seam ROI point cloud segmentation. In the image segmentation stage, an enhanced segmentation network is constructed by integrating a convolutional attention module into YOLOv8n-seg, which effectively improves the localization accuracy and mask extraction quality of the weld seam region. In the point cloud segmentation stage, the 3D point cloud is first mapped onto a 2D pixel plane to achieve spatial alignment. Subsequently, a coarse screening of the projected point cloud is performed based on the bounding boxes output from the instance segmentation, eliminating a large amount of redundant data. Furthermore, a grayscale matrix is constructed based on the segmentation masks, enabling precise extraction of the weld seam ROI point cloud through point-wise discrimination. Experimental results demonstrate that the proposed method achieves high-quality segmentation of the weld seam region, providing a reliable foundation for robotic automated grinding.

## 1. Introduction

To ensure the mechanical properties of welded structural components, weld grinding has become a critical process in the equipment manufacturing industry. With the advancement of industrial technology, weld grinding has gradually evolved from traditional manual operations to robotic intelligent grinding [1]. Robotic intelligent grinding typically employs vision sensors to detect and segment the weld target region (ROI), followed by grinding trajectory planning to guide industrial robots in efficient operations. Among these, weld ROI detection and segmentation are considered the key and essential prerequisites for robotic intelligent grinding [2]. Compared to general objects such as tables, chairs, vehicles, or humans, welds exhibit smaller color differences from the background, making weld ROI detection and segmentation more sensitive to lighting conditions. Additionally, post-weld seams are characterized by irregular, diverse, and random shapes, which have made rapid weld ROI detection and precise segmentation persistent core challenges in robotic intelligent grinding.

In robotic grinding systems, visual sensors are generally categorized into passive and active vision [3]. Passive vision utilizes industrial CCD cameras to capture images of workpieces under natural light, followed by image processing to extract seam ROIs [4]. With the development of deep learning, some classic object detection and segmentation algorithms have been applied to weld seam ROI extraction. Li et al. [5] employed Mask R-CNN for instance segmentation of weld seams; Wang et al. [6] proposed an improved algorithm based on YOLOv5n for weld seam detection and trajectory extraction. Tung et al. [7] developed an automated robotic cleaning system by detecting and segmenting weld seam ROI using Mask R-CNN. Natan et al. [8] enhanced the U-Net model to segment welding points of workpieces by capturing image or video frames. Additionally, several studies [9,10,11,12,13] have utilized traditional object detection algorithms to detect weld seam boundaries, textures, and other features for seam ROI segmentation. However, 2D images lack depth information, resulting in extracted seam ROIs that do not accurately represent the true seam morphology for precise grinding path generation. These methods are generally suitable only for simple planar seam grinding scenarios or quality inspection tasks.

By projecting structural light or laser to the surface of the weld, active vision can stably capture the three-dimensional point cloud data of the weld, making up for the lack of depth information expression of two-dimensional images. Nevertheless, point clouds are typically disordered, sparse, and unevenly distributed, which severely affects the accuracy of feature extraction and target localization [14]. Therefore, early methods relied on manual processing of seam ROIs to construct precise 3D models of workpieces [15,16,17], followed by offline trajectory planning in programming environments.These approaches are inefficient, subjective, and limited to batch grinding tasks for standardized workpieces.To enhance weld ROI detection and segmentation efficiency, Ge et al. [18] developed weld tracking software and proposed and constructed an online automatic grinding system for welds based on laser vision sensors. In subsequent research [19], they utilized a model-based segmentation method for weld extraction, converting spatial point clouds into two-dimensional point clouds, and then performed weld segmentation based on the least squares method with threshold comparison, significantly improving processing speed and accuracy. Liu et al. [20] proposed a method for determining the cutting plane based on 3D projection and image binarization, which was used to extract weld feature points and automate the planning of grinding paths on the weld surface, thereby enhancing grinding efficiency. Meng et al. [21] proposed an improved B-spline curve weld grinding trajectory planning method integrating point cloud segmentation, feature point removal, point cloud light smoothing, and hole repair. However, these methods depend on the regular geometric features and statistical principles of workpieces, making them suitable for handling single-structured scenarios. They often struggle to adapt to complex and diverse seam types, resulting in suboptimal ROI extraction. In recent years, 3D point cloud instance segmentation based on deep learning has garnered widespread attention, enabling autonomous analysis and processing of captured point cloud data to accurately identify seam positions and shapes, achieving end-to-end output [22]. For example, Pandiyan et al. [23] utilized advanced network models and intelligent control systems to achieve automatic detection of weld endpoints during robotic belt grinding. Zhan et al. [24] proposed a weld instance segmentation method based on RGB-D level feature fusion, expanded the RGB-D-level feature fusion module based on the Mask R-CNN feature pyramid network (FPN), and added MaskGIoU Head to improve the segmentation accuracy of weld positioning. Dong et al. [25] introduced a 1D convolutional neural network for autonomous identification and segmentation of grinding points for weld grinding. Nonetheless, existing deep learning models face challenges in processing large-scale point cloud data, including long training times, high computational resource consumption, and limited generalization capabilities when dealing with complex or irregular weld morphologies.

In summary, the efficient detection and accurate segmentation of weld ROI constitute a highly challenging 3D data understanding task, with the core difficulties lying in the fact that the weld morphology exhibits both diversity (varying forms under the same semantic label) and randomness (no clear geometric boundary with the base material). To address these challenges, this paper proposes a method for weld ROI detection and segmentation based on the fusion of active and passive vision. By integrating dual-modal data of point clouds and images, the method achieves comprehensive scene perception to compensate for the limitations of a single modality, realizes efficient pixel-level point cloud segmentation of weld ROI, and thus provides prerequisites and technical support for intelligent robotic grinding.

## 2. The Proposed Method

**Figure 1:**
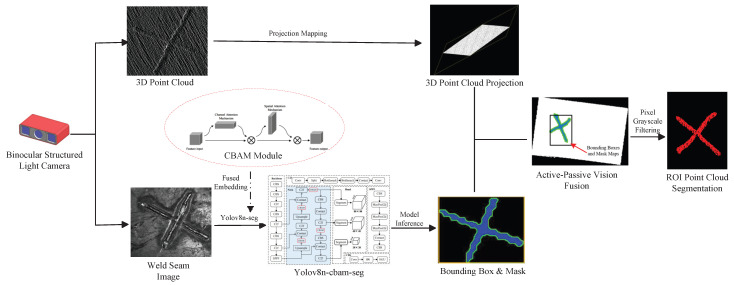
Framework for weld seam ROI detection and segmentation method based on active–passive vision fusion.

This paper utilizes a binocular structured-light camera to acquire two modal data types of the workpiece—2D images and 3D point clouds—and proposes a weld seam detection and segmentation method based on the fusion of active and passive vision. As illustrated in Figure 1, the proposed approach mainly consists of two steps: weld seam image instance segmentation and weld seam ROI point cloud segmentation. In the weld seam image instance segmentation stage, to enhance the localization accuracy of the weld seam region and the extraction quality of the mask map, an improved segmentation algorithm is designed by integrating the convolutional block attention module (CBAM) into the YOLOv8n-seg model. In the weld seam ROI point cloud segmentation stage, the 3D point cloud is first mapped onto the 2D pixel plane using a calibrated parameter matrix to achieve spatial alignment between point cloud coordinates and pixel coordinates. Subsequently, using the weld seam ROI bounding boxes output from the instance segmentation stage as boundaries, a coarse screening of the projected point cloud is performed to rapidly eliminate a large number of redundant point clouds in non-weld seam regions. Finally, a fine screening operation is executed: a grayscale matrix is constructed based on the high-precision mask maps generated by the instance segmentation, and point-wise discrimination is applied to the coarsely screened point cloud to further remove interfering points outside the mask region, ultimately achieving precise segmentation of the weld seam ROI point cloud.

### 2.1. Instance Segmentation of Weld Seam Images

**Figure 2:**
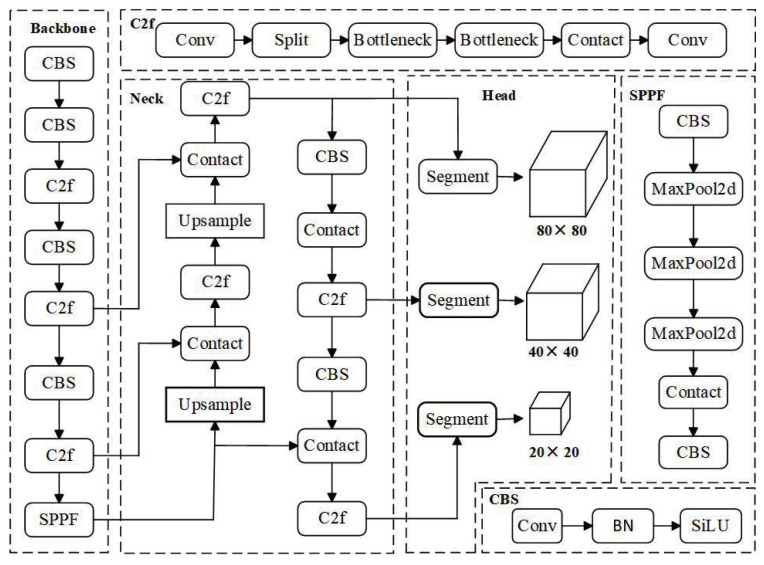
YOLOv8n-seg network architecture.

YOLOv8, part of the YOLO model family, is designed for tasks such as object detection, instance segmentation, and classification. YOLOv8n-seg is the most “lightweight” model in this series for instance segmentation, requiring minimal parameters and computational resources. The network structure of the YOLOv8n-seg model [26] consists of the following components, as illustrated in Figure 2: input layer, backbone network, neck network, and output head. The backbone is composed of modules such as CBS convolution, C2f, and SPPF, arranged sequentially to extract multi-scale and multi-category instance features from images. The neck network adopts the classical PAFPN architecture [27] from YOLOv5, ensuring robust feature fusion across scales. The output head provides both object detection bounding boxes and segmentation mask information.

**Figure 3:**
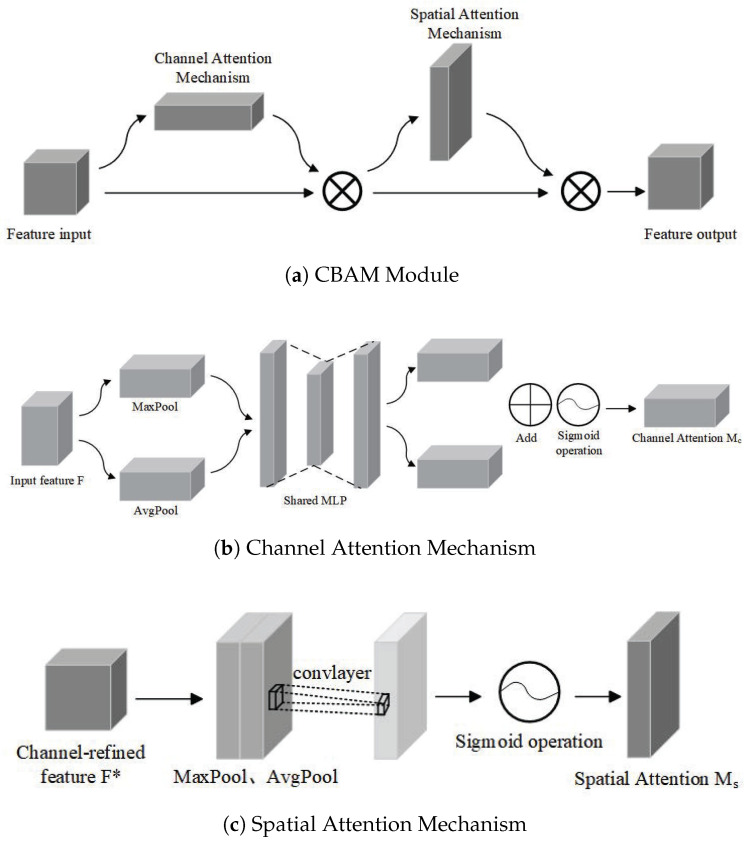
Schematic diagram of the convolutional block attention module (CBAM) structure.

Compared to previous YOLO series algorithms, YOLOv8n-seg demonstrates superior detection speed and accuracy. However, its segmentation performance still shows limitations when dealing with complex scenes where the target and background are highly similar. To address this challenge, this study introduces the convolutional block attention module (CBAM), which can automatically assign high weights to critical information while disregarding redundant data with lower weight, thereby enhancing the model’s sensitivity to the weld ROI. CBAM consists of two components, the channel attention mechanism and the spatial attention mechanism, as shown in Figure 3. These two mechanisms work in synergy; they not only strengthen the feature representation ability across different channels but also accurately highlight key information in spatial positions, achieving effective distinction and precise segmentation between the target and background.

The channel attention mechanism (CAM) initially performs max pooling and average pooling operations on the input feature map, then feeds them into a multiLayer perceptron (MLP) to learn the feature weights of each channel, which are then summed. The channel attention feature weight matrix $M_{c}$ is then output via a sigmoid activation function. The MLP consists of two linear layers and a ReLU activation function. The channel attention mechanism can be formulated as follows:

(1) $$M_{c}\left(F\right)=\sigma \left\{\mathrm{MLP}\left[\mathrm{AvgPool}(F)\right]+\mathrm{MLP}\left[\mathrm{MaxPool}(F)\right]\right\}$$

where F is the input feature map; MLP denotes the multilayer perceptron operation; AvgPool and MaxPool represent the global max pooling and global average pooling operations on the channel dimension for F, respectively; and σ represents the Sigmoid activation function.

The spatial attention mechanism (SAM) first performs channel-based global max pooling and average pooling operations, followed by concatenation along the channel dimension. A 7 × 7 convolution operation is then applied to the concatenated feature map for further processing, generating a feature map with a channel dimension of 1. Finally, the Sigmoid activation function generates the spatial attention feature weight matrix $M_{s}$. The spatial attention mechanism can be formulated as follows:

(2) $$M_{s}\left(F\right)=\sigma \left\{f^{7\times 7}\left[\mathrm{AvgPool}\left(F\right);\mathrm{MaxPool}\left(F\right)\right]\right\}$$

where $f^{7\times 7}$ is a convolution operation that is performed.

**Figure 4:**
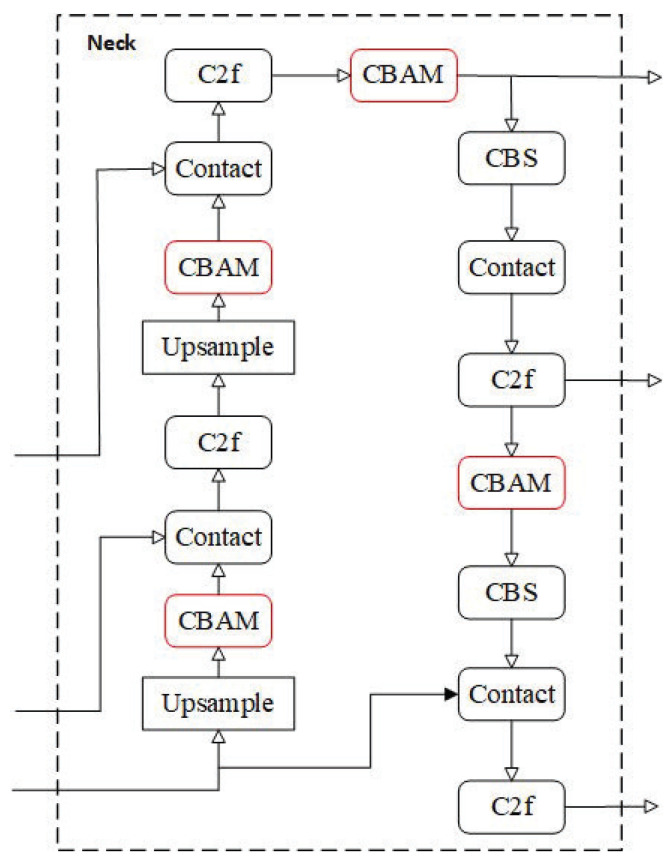
Neck architecture in YOLOv8n-cbam-seg.

To enhance the model’s capability in perceiving and distinguishing weld seam features within complex industrial scenarios, this study integrates the convolutional block attention module (CBAM) into the neck component (comprising the feature pyramid network and path aggregation network) of the YOLOv8n-seg architecture. As illustrated in Figure 4, within the neck structure, the CBAM modules are incorporated following each C2f module and are interactively coupled with operations such as Upsample and Concat. This design enables the model to refine multi-scale feature representations during the feature fusion process in the neck, thereby directing subsequent feature processing to focus more effectively on critical weld seam characteristics. Consequently, this enhancement improves the accuracy of subsequent object detection and instance segmentation tasks. The modified model is designated as YOLOv8n-cbam-seg.

### 2.2. Weld ROI Point Cloud Segmentation

To achieve spatial alignment between 3D point clouds and 2D images while establishing a unified coordinate system for subsequent weld seam point cloud segmentation, this paper proposes an active–passive vision fusion approach. Specifically, based on the calibration parameters of the binocular structured-light camera, a projective mapping model is established. As shown in Equation (3), this model projects 3D point clouds from the world coordinate system onto a 2D pixel plane:

(3) $$\left[\begin{array}{c} u_{i}\\ v_{i}\\ 1 \end{array}\right]=\beta M_{1}M_{2}\left[\begin{array}{c} X_{i}\\ Y_{i}\\ Z_{i}\\ 1 \end{array}\right]$$

where $\left(X_{i},Y_{i},Z_{i}\right)$ represents the coordinates of any point $p_{i}$ in the point cloud, β is the coordinate normalization coefficient, $M_{1}$ and $M_{2}$ are the camera’s intrinsic and extrinsic parameter matrices, and $\left(u_{i},v_{i}\right)$ denotes the pixel coordinates of point $p_{i}$, where $i\in N^{+}$ is the point index.

**Figure 5:**
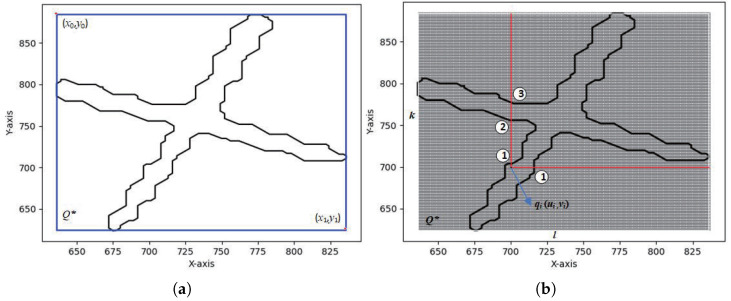
ROI point selection. (**a**) Coarse Screening of Detection Boxes; (**b**) Fine Screening by Pixel Intensity.

Let the set of transformed point cloud pixels be denoted as Q, and the set of weld ROI mask contours output by YOLOv8n-cbam-seg be denoted as S. For a point $p_{i}$, its pixel coordinates are $q_{i}\in Q$. If $q_{i}$ is inside S, then $p_{i}$ belongs to the weld ROI point cloud; otherwise, it does not. In a two-dimensional plane, the “ray casting method” [28] is typically used to determine the relationship between a point and a region. However, this method requires traversing all pixel points in Q and performing complex geometric computations to find the intersection of the “ray” with the contour S, which leads to slow segmentation efficiency for weld seam ROI point clouds. To address this issue, this paper proposes a new pixel grayscale filtering method, which is mainly divided into three steps:

1. Coarse filtering. Based on the weld detection bounding box information $\left(x_{0},y_{0},x_{1},y_{1}\right)$ output by YOLOv8n-cbam-seg, where $\left(x_{0},y_{0}\right)$ is the top-left pixel coordinate and $\left(x_{1},y_{1}\right)$ is the bottom-right pixel coordinate, the pixels in Q that are covered by the detection box are filtered out and denoted as $Q^{*}$, as shown in Figure 5a.

2. Grayscale matrix construction. Based on the detection box $\left(x_{0},y_{0},x_{1},y_{1}\right)$, a pixel grayscale matrix $L_{p}$ is constructed. The length l and width w of the matrix are determined as follows:

(4) $$\begin{array}{c} l=\left\lceil \left(\frac{x_{1}-x_{0}}{p_{x}}\right)\right\rceil \\ w=\left\lceil \left(\frac{y_{1}-y_{0}}{p_{y}}\right)\right\rceil \end{array}$$

where ⌈⌉ denotes the ceiling operator, and $p_{x}$ and $p_{y}$ are the pixel dimensions in the X and Y directions respectively. The elements of the $L_{p}$ matrix are initialized with a default value of 255. Denoting the set of mask contours output by YOLOv8n-cbam-seg as *S*, where the coordinates of any mask point $S_{k}\in S$ are given by $\left(x_{k},y_{k}\right)$, its corresponding position in the $L_{p}$ matrix is determined by the following equation:

(5) $$\begin{array}{cc} l_{k} & =\left\lceil \frac{x_{k}-x_{0}}{p_{x}}\right\rceil \\ w_{k} & =\left\lceil \frac{y_{k}-y_{0}}{p_{y}}\right\rceil \end{array}$$

Thus, the point $S_{k}$ is located at row $l_{k}$ and column $w_{k}$ in the $L_{p}$ matrix, and the grayscale value at this position is updated to 0.

3. Fine filtering. For any pixel point $q_{i}=\left(u_{i},v_{i}\right)$ in $Q^{*}$, the corresponding position in the $L_{p}$ matrix is calculated as follows:

(6) $$\begin{array}{cc} l_{i} & =\left\lceil \frac{u_{i}-x_{0}}{p_{x}}\right\rceil \\ w_{i} & =\left\lceil \frac{v_{i}-y_{0}}{p_{y}}\right\rceil \end{array}$$

From the position $\left(l_{i},w_{i}\right)$, a ray is drawn in both the horizontal and vertical directions. The number of pixels with a grayscale value of 0 in each ray within the $L_{p}$ matrix is recorded as $C_{i}$. According to the ray-casting method, if $C_{i}$ is odd for any ray, the pixel point $q_{i}$ corresponds to a point $p_{i}$ that belongs to the weld seam ROI region; otherwise, it does not, as shown in Figure 5b.

By iterating through all pixel points in $Q^{*}$ using the aforementioned procedure, pixel-level precise screening of the weld seam ROI point cloud can be achieved.

## 3. Experimental Analysis

### 3.1. Data Collection and Processing

**Figure 6:**
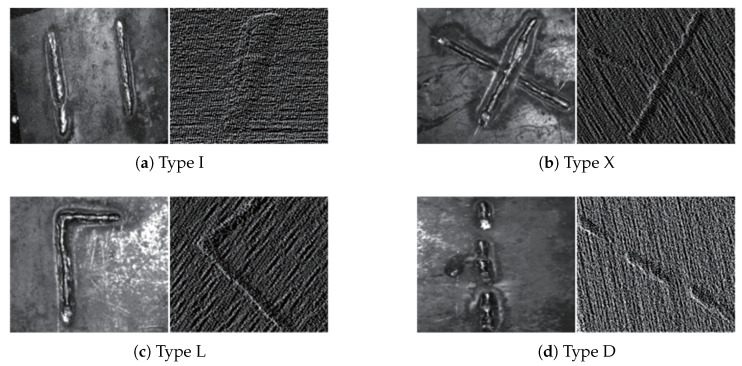
Experimental weld specimens.

This study employs an Elson binocular line-scan structured-light camera with a baseline distance of 200 mm and a resolution of 1920 × 1200 pixels. System calibration was performed using Zhang’s calibration method in conjunction with a standard checkerboard, achieving a mean reprojection error of 0.15 ± 0.03 pixels. Data were acquired at a typical working distance of 600 mm, with the system covering a field of view of 640 × 400 mm. As shown in Figure 6, four types of small-target overlay weld specimens were designed—I-shaped, X-shaped, L-shaped, and three-segment discontinuous D-shaped—yielding a total of 40 randomly combined weld seam samples. Each scanned dataset includes a 2D image and its corresponding 3D point cloud. Diverse data were obtained by varying lighting conditions, shooting distance, angle, workpiece type, and position to simulate complex industrial scenarios. The initially collected 300 weld seam images were augmented to 1800 via flipping, affine transformation, brightness adjustment, blurring, and Gaussian noise injection. All images were pixel-wise mask-annotated by experienced engineers using the LabelMe tool, with contours strictly traced along the weld seams to ensure consistency.

The experimental platform operated on Ubuntu 18.04.6 LTS, with a 13th Gen Intel^®^ Core™ i5-13400F CPU (Intel, Santa Clara, CA, USA), an NVIDIA GeForce RTX 3060Ti GPU, and 32 GB of RAM (NVIDIA, Santa Clara, CA, USA). The deep learning framework was built on PyTorch 1.12, compatible with Python 3.8.16, utilizing CUDA version 11.3 and cuDNN version 8.9.2 for GPU acceleration. The dataset of 1800 images was pre-divided into training, validation, and test sets in a 6:2:2 ratio. To ensure unbiased evaluation, data augmentation techniques—such as flipping and brightness adjustment—were applied exclusively to the training set, thereby preserving the purity of the validation and test sets and preventing data leakage. After augmentation, the training, validation, and test sets comprised 1080, 360, and 360 images, respectively. Detailed hyperparameter configurations for model training are summarized in Table 1.

### 3.2. Data Collection and Processing

#### 3.2.1. Model Training Results

**Figure 7:**
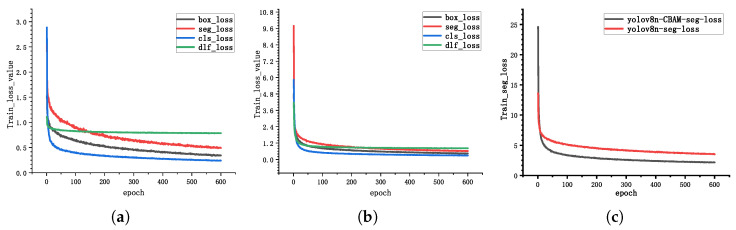
A comparison of training loss curves between YOLOv8n-seg and the proposed YOLOv8n-cbam-seg model. (**a**) YOLOv8n-seg network training loss value; (**b**) YOLOv8n-cbam-seg network training loss value; (**c**) The comprehensive loss value of each model.

Figure 7 shows the loss function convergence curves during the training process for both YOLOv8n-seg and YOLOv8n-cbam-seg networks. Here, box_loss represents the bounding box loss, seg_loss represents the segmentation loss, cls_loss represents the classification loss, and dfl_loss represents the distribution focal loss. The x-axis represents the number of epochs, indicating the number of times the samples have been traversed. As shown in Figure 7a,b, during the initial training phase, the values of all loss functions decrease rapidly, indicating that the model is quickly fitting the training data. YOLOv8n-seg begins to converge after 100 epochs, while YOLOv8n-cbam-seg demonstrates better convergence after just 50 epochs. Figure 7c plots the sum of the loss values of each of the two models, showing that the YOLOv8n-cbam-seg network has a smaller loss value, indicating a better fit to the data.

**Figure 8:**
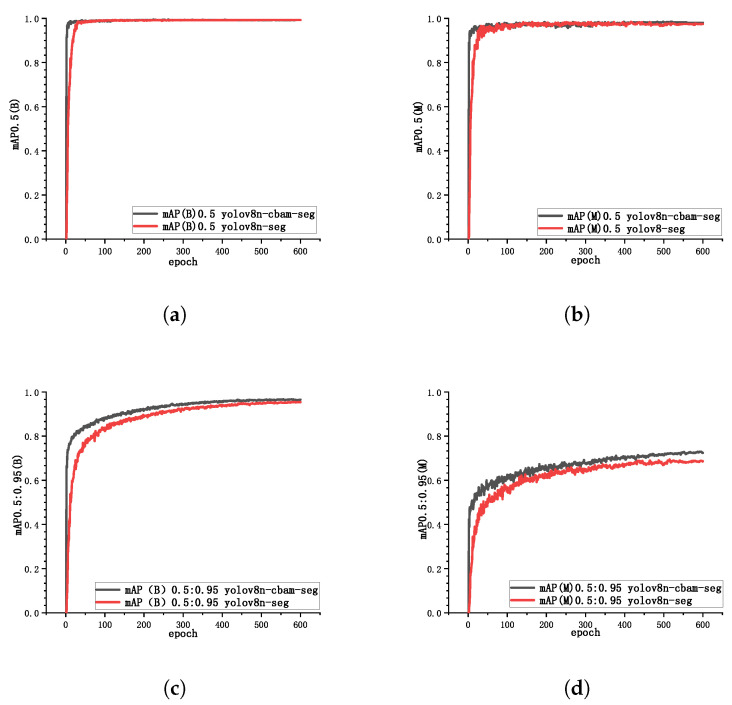
Variation curves of mAP values for different categories. (**a**) mAP0.5(B) value; (**b**) mAP0.5(M) value; (**c**) mAP0.5:0.95(B) value; (**d**) mAP0.5:0.95(M) value.

Figure 8 presents the mean average precision (mAP) metrics for both YOLOv8n-seg and YOLOv8n-cbam-seg networks at different intersection over union (IoU) thresholds, used to evaluate the overall performance of the models. Specifically, mAP0.5(B) and mAP0.5(M) represent the average precision of bounding boxes (B) and instance segmentation masks (M) at IoU = 0.5, respectively, while mAP0.5:0.95(B) and mAP0.5:0.95(M) provide a more comprehensive assessment of model localization and segmentation performance across multiple IoU thresholds. Experimental results show that compared to YOLOv8n-seg, YOLOv8n-cbam-seg demonstrates faster convergence in all four metrics and achieves higher precision in mAP0.5:0.95(B) and mAP0.5:0.95(M). The precision in mAP0.5(B) and mAP0.5(M) for both models is comparable. The results validate that the introduction of CBAM enhances both detection speed and segmentation accuracy in the YOLOv8n-cbam-seg network.

**Figure 9:**
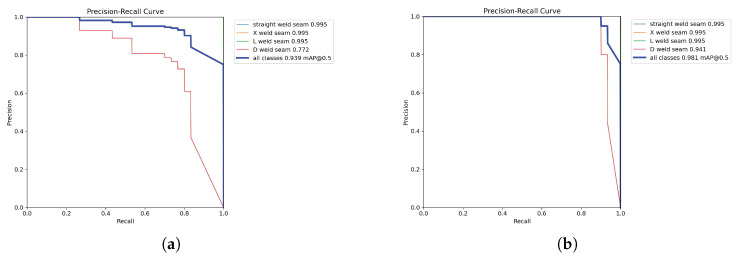
Comparison of precision–recall curves. (**a**) Mask-PR Curve of YOLOv8n-seg; (**b**) Mask-PR Curve of YOLOv8n-cbam-seg.

To further analyze model performance across different decision thresholds, Figure 9 presents the precision–recall (PR) curves of both models. It can be observed that the PR curve of YOLOv8n-CBAM-seg lies closer to the upper-right corner of the coordinate plane and exhibits a larger area under the curve (AUC) across all categories, indicating its ability to maintain a superior balance between precision and recall under varying confidence thresholds. Notably, for the D-shaped weld seams—characterized by complex geometry and high segmentation difficulty—the proposed model achieves a 16.9% improvement in average precision (AP) compared to the baseline. This enhancement, coupled with the capability of the PR curve to sustain high precision at high recall rates, is particularly critical for industrial applications such as weld inspection where missed detections are unacceptable. It demonstrates that the model ensures the identification of all actual weld seams while effectively reducing the risk of false positives.

#### 3.2.2. Ablation Studies and Analysis

To quantitatively evaluate the contribution of the introduced CBAM module and its individual components to model performance, we designed systematic ablation studies. All experiments were conducted under the same dataset and training configuration described in Section 3.1 to ensure fair comparability. We first compared the computational efficiency between the baseline model and the enhanced model, with results summarized in Table 2. Although the incorporation of CBAM introduces a modest increase in parameters and computational load, the resulting performance gains substantially outweigh this minor computational cost.

The results of the ablation studies are presented in Table 3. Using the standard YOLOv8n-seg as the baseline (A), we sequentially integrated the channel attention module (CAM) and spatial attention module (SAM) into the architecture.

The experimental results demonstrate the following: (1) The individual integration of either attention module consistently improves performance: both Model B (with CAM only) and Model C (with SAM only) surpass the baseline (Model A) across all evaluation metrics. This confirms the effectiveness of feature recalibration in either channel or spatial dimensions. (2) Spatial attention exhibits more pronounced contributions: Model C generally achieves higher performance gains than Model B, potentially because the local textural and structural differences between weld seams and background regions are more critical than overall channel-wise distribution disparities. (3) Synergistic effect of CBAM: The final model (Model D), integrating both CAM and SAM, achieves optimal performance across all metrics. This verifies the complementary nature of channel and spatial attention mechanisms, where their coordinated operation comprehensively enhances feature representation, thereby yielding the most accurate results for both object detection and instance segmentation tasks.

### 3.3. Weld ROI Point Cloud Segmentation Results

**Figure 10:**
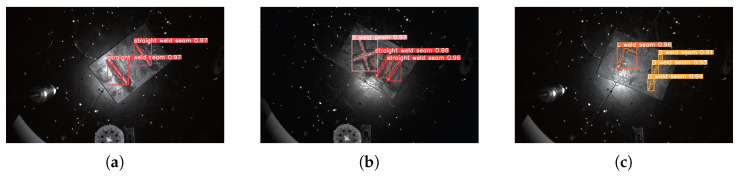
Weld seam ROI image segmentation results based on YOLOv8n-cbam-seg. (**a**) Experimental Specimen 1; (**b**) Experimental Specimen 2; (**c**) Experimental Specimen 3.

In this study, three experimental workpieces were used for weld seam ROI point cloud segmentation. The input data consisted of 2D images and 3D point clouds obtained from a binocular line-scan structured light camera. Following the proposed method in this paper, the YOLOv8n-cbam-seg network, trained in the previous section, was used for weld seam ROI image segmentation. The segmentation results are shown in Figure 10. As seen in the figure, YOLOv8n-cbam-seg accurately identified the weld seam detection boxes and segmentation masks.

**Figure 11:**
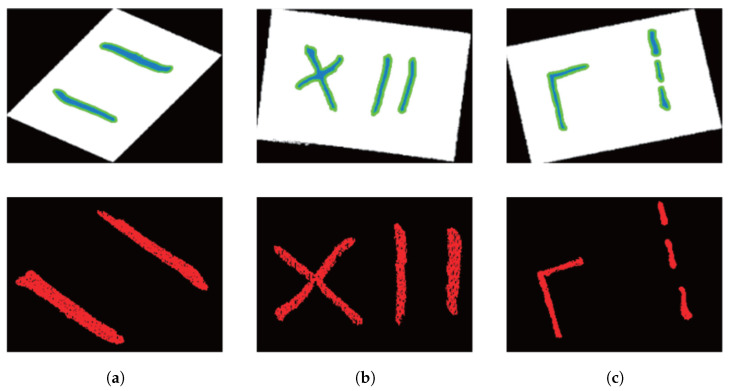
Weld seam ROI point cloud segmentation results based on pixel grayscale screening. (**a**) Experimental Specimen 1; (**b**) Experimental Specimen 2; (**c**) Experimental Specimen 3.

The 3D point cloud is projected onto the pixel plane to achieve main passive vision fusion, followed by point cloud segmentation based on the weld seam ROI mask. The segmentation results are shown in Figure 11. The upper row shows the active and passive visual fusion results, where the white dots represent the point cloud projected onto the pixel plane and the colored dots represent the weld ROI mask segmented by the instance. The lower line is the result of weld ROI point cloud segmentation. The experimental results demonstrate that the proposed method effectively identifies and segments the weld seam ROI point clouds across all three workpieces.

### 3.4. Robot Weld Grinding Experiment

**Figure 12:**
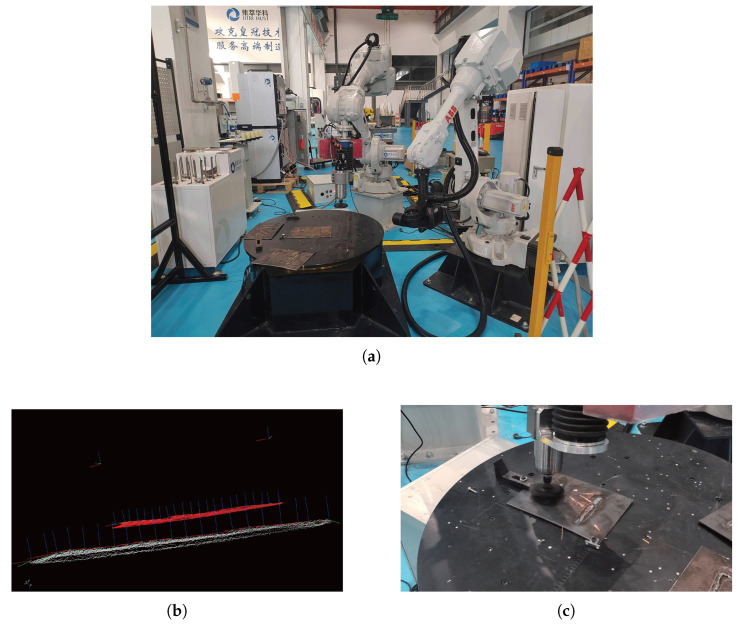
Robotic weld grinding. (**a**) Grinding Experimental Platform; (**b**) Grinding Trajectory; (**c**) Weld Grinding.

This study established an integrated weld seam grinding experimental platform comprising an ABB IRB2600-20/165 robot, a binocular line-structured-light camera, and an end-effector force-controlled grinding head. The vision system was configured in an “eye-in-hand” arrangement (Figure 12a). Based on the accurately segmented weld seam ROI point cloud obtained through the proposed method, grinding trajectories were generated using the slicing method [29] (Figure 12b). Under the process parameters of spindle speed 12,000 rpm, normal force 15 N, and feed rate 60 mm/s (Figure 12c), quantitative evaluation demonstrated that the average deviation between the actual grinding trajectory and the planned path was 0.32 mm; the surface roughness Ra of the weld seam was reduced from 12.5 μm before grinding to 3.2 μm; and the total cycle time for a single operational task from perception to grinding completion was 48 s. These results fully validate the capability of the proposed method to achieve autonomous weld seam identification and precision grinding, effectively enhancing the intelligence level of robotic grinding operations.

## 4. Conclusions

This paper addresses the challenge of rapid detection and precise segmentation of multi-type weld seams in robotic intelligent grinding by proposing an active–passive vision fusion-based method for weld seam ROI detection and point cloud segmentation. The core of this approach lies in integrating 2D image and 3D point cloud data to establish an integrated processing pipeline from visual perception to trajectory planning for weld seam grinding. The main contributions can be summarized as follows: (1) An improved YOLOv8n-CBAM-seg instance segmentation network is proposed, where the incorporation of the convolutional block attention module (CBAM) effectively enhances the model’s capability to represent weld seam features and improves segmentation accuracy under complex backgrounds. (2) An efficient active vision fusion processing method is designed, achieving accurate identification and rapid segmentation from 2D weld seam ROI masks to 3D point clouds, thereby reducing the complexity associated with direct 3D point cloud processing. (3) A comprehensive robotic intelligent grinding system is constructed, and multiple quantitative metrics—including trajectory deviation, surface roughness, and operational cycle time—collectively demonstrate the method’s effectiveness, precision, and efficiency in practical applications. However, this study currently focuses primarily on planar and simple geometric weld seams, and its generalizability to complex curved configurations (such as saddle-shaped and circular welds) requires systematic validation. Future work will aim to expand the scale and diversity of the dataset by incorporating more weld seam types in complex 3D spaces, and further explore network architectures and segmentation strategies with enhanced robustness to complex geometries, thereby improving the method’s universality and industrial application potential.

## Figures and Tables

**Table 1:** Model training parameter settings.

Parameter	Value	Parameter	Value
Training Epochs	600	Optimizer	AdamW
Initial Learning Rate	0.01	Input Size	1920 × 1200
Batch Size	16	Decay Coefficient	0.0005

**Table 2:** Comparison of model computational complexity.

Model	Params (M)	GFLOPs	FPS
YOLOv8n-seg (Baseline)	3.25	12.0	156
YOLOv8n-CBAM-seg (Ours)	3.42	14.1	148

**Table 3:** Ablation study on CBAM.

Model	CAM	SAM	BBox mAP (%)		Mask mAP (%)	
			@0.5	@0.5:0.95	@0.5	@0.5:0.95
A: Baseline	–	–	96.2	68.5	95.8	67.1
B: Baseline + CAM	✓	–	96.8	70.1	96.3	68.9
C: Baseline + SAM	–	✓	97.1	71.3	96.7	70.2
D: Baseline + CBAM (Ours)	✓	✓	97.5	72.6	97.2	71.5

## Data Availability

The data that support the findings of this study are available upon reasonable request from the corresponding author. However, the data are not publicly available due to privacy or ethical restrictions.

## References

[1] Huynh H.N., Assadi H., Rivière-Lorphèvre E., Verlinden O., Ahmadi K. (2020). Modelling the dynamics of industrial robots for milling operations. Robot. Comput.-Integr. Manuf..

[2] Guo W., Huang X., Qi B., Ren X., Chen H., Chen X. (2024). Vision-guided path planning and joint configuration optimization for robot grinding of spatial surface weld beads via point cloud. Adv. Eng. Inform..

[3] Wang N., Zhong K., Shi X., Zhang X. (2020). A robust weld seam recognition method under heavy noise based on structured-light vision. Robot. Comput. Integr. Manuf..

[4] Huang K., Dong Z., Wang J., Fei Y. (2023). Weld bead segmentation using RealSense depth camera based on 3D global features and texture features of subregions. Signal Image Video Process..

[5] Li J., Li B., Dong L., Wang X., Tian M. (2022). Weld seam identification and tracking of inspection robot based on deep learning network. Drones.

[6] Wang J., Yao J., Liu X., Du Y., Liu M., Su Y., Lu D. (2024). Weld Detection and Tracking Algorithm for Inspection Robot Based on Deep Learning. Proceedings of the 2024 International Conference on Electronic Engineering and Information Systems (EEISS).

[7] Tung T.-J., Al-Hussein M., Martinez P. (2023). Vision-Based Guiding System for Autonomous Robotic Corner Cleaning of Window Frames. Buildings.

[8] Natan O., Putri D.U.K., Dharmawan A. (2021). Deep learning-based weld spot segmentation using modified UNet with various convolutional blocks. ICIC Express Lett. Part B Appl..

[9] Haffner O., Kučera E., Kozák Š. (2016). Weld segmentation for diagnostic and evaluation method. Proceedings of the 2016 Cybernetics & Informatics (K&I).

[10] Malarvel M., Sethumadhavan G., Bhagi P.C.R., Kar S., Thangavel S. (2017). An improved version of Otsu’s method for segmentation of weld defects on X-radiography images. Optik.

[11] Wang X., Yang Y., Kong J., Shi Y. (2019). Image Binarization Method of Equal-thickness Butt Welds Based on Regional Optimization. China Mech. Eng..

[12] Li X., Li X., Ge S.S., Khyam M.O., Luo C. (2017). Automatic welding seam tracking and identification. IEEE Trans. Ind. Electron..

[13] Golodov V.A., Maltseva A.A. (2022). Approach to weld segmentation and defect classification in radiographic images of pipe welds. NDT E Int..

[14] Fei B., Yang W., Chen W.-M., Li Z., Li Y., Ma T., Hu X., Ma L. (2022). Comprehensive review of deep learning-based 3d point cloud completion processing and analysis. IEEE Trans. Intell. Transp. Syst..

[15] Wang X., Zhang X., Ren X., Li L., Feng H., He Y., Chen H., Chen X. (2020). Point cloud 3D parent surface reconstruction and weld seam feature extraction for robotic grinding path planning. Int. J. Adv. Manuf. Technol..

[16] Jing O., Lai Z., Qinghong W., Xin L., Yingjie L. (2021). Weld-seam identification and model reconstruction of remanufacturing blade based on three-dimensional vision. Adv. Eng. Inform..

[17] Wilson J.M., Piya C., Shin Y.C., Zhao F., Ramani K. (2014). Remanufacturing of turbine blades by laser direct deposition with its energy and environmental impact analysis. J. Clean. Prod..

[18] Ge J., Deng Z., Li Z., Li W., Lv L., Liu T. (2021). Robot welding seam online grinding system based on laser vision guidance. Int. J. Adv. Manuf. Technol..

[19] Ge J., Deng Z., Li Z., Li W., Liu T., Zhang H., Nie J. (2022). An efficient system based on model segmentation for weld seam grinding robot. Int. J. Adv. Manuf. Technol..

[20] Liu Y., Yang S., Tang Q., Tian X. (2024). A novel path planning method of robotic grinding for free-form weld seam based on 3D point cloud. Int. J. Adv. Manuf. Technol..

[21] Meng Y., Jiang Y., Li Y., Pang G., Tong Q. (2023). Research on point cloud processing and grinding trajectory planning of steel helmet based on 3D scanner. IEEE Access.

[22] Guo Y., Wang H., Hu Q., Liu H., Liu L., Bennamoun M. (2020). Deep learning for 3d point clouds: A survey. IEEE Trans. Pattern Anal. Mach. Intell..

[23] Song Y., Yang H., Lv H. (2012). Intelligent control for a robot belt grinding system. IEEE Trans. Control Syst. Technol..

[24] Zhan S., Qian K., Liu Y., Gong Y. (2022). Weld Seam Segmentation in RGB-D Data using Attention-based Hierarchical Feature Fusion. Proceedings of the 2022 China Automation Congress (CAC).

[25] Dong C., Shi T., Zhao Q., Huang X., Liu C. (2021). 1D Segmentation Network for 3D Seam Weld Grinding. J. Phys. Conf. Ser..

[26] Varghese R., Sambath M. (2024). YOLOv8: A Novel Object Detection Algorithm with Enhanced Performance and Robustness. Proceedings of the 2024 International Conference on Advances in Data Engineering and Intelligent Computing Systems (ADICS).

[27] Jocher G., Chaurasia A., Stoken A., Borovec J., Kwon Y., Michael K., Fang J., Wong C., Yifu Z., Montes D. (2022). ultralytics/yolov5: V6. 2-yolov5 classification models, apple m1, reproducibility, clearml and deci. ai integrations. Zenodo.

[28] Wang W.C., Wu E.H. (2000). A New Method for Deciding Whether a Point is in a Polygon or a Polyhedron. J. Softw..

[29] Chen W., Li X., Ge H., Wang L., Zhang Y. (2020). Trajectory planning for spray painting robot based on point cloud slicing technique. Electronics.

